# Prevalence of *Chlamydia trachomatis* and *Mycoplasma genitalium* in pregnant women of Sabzevar-Iran

**Published:** 2011-09

**Authors:** M Haghighi Hasanabad, M Mohammadzadeh, A Bahador, N Fazel, H Rakhshani, A Majnooni

**Affiliations:** 1 Molecular and Cellular Biology Research Center, School of Medicine, Sabzevar University of Medical Sciences, Sabzevar, Iran; 2 Departments of Microbiology, School of Medicine, Tehran University of Medical Sciences, Tehran, Iran; 3 Research center of Infectious and Tropical Disease, Tabriz University of Medical Sciences, Tabriz, Iran

**Keywords:** Prevalence, *Chlamydia trachomatis*, *Mycoplasma genitalium*, Pregnant women

## Abstract

**Background:**

As prenatal screening for sexually transmitted infections and treatment of infected pregnant women is not routinely performed in Iran and prevalence of two sexually transmitted pathogens, *Chlamydia trachomatis* and *Mycoplasma genitalium*, in Sabzevar (east of Iran) is unknown, we decided to perform this prospective study.

**Methods:**

One hundred ninety-six urine specimens of pregnant women attending the specialized maternity hospital of the city were collected and tested by duplex PCR.

**Results:**

A total of 31 specimens were positive (15.81%) (27 *Chlamydia trachomatis* isolates, 13.77%; and 2 *Mycoplasma genitalium* isolates, 1.02%). Co-infection with both species was detected in 2 specimens (1.02%). A significant correlation was found between preterm labor and infection (P-value ≤ 0.05).

**Conclusion:**

The present study shows high prevalence of Chlamydial infections in comparison with *Mycoplasma genitalium* in this region. Further studies with larger sample size and more focused on different groups at risk are needed for a movement towards prevention and control of sexually transmitted infections (STIs).

## INTRODUCTION

There are more than 30 different sexually transmissible agents and the most common treatable and preventable of them is *Chlamydia trachomatis*. Rapid detection of Chlamydial infections in the medical laboratory is very important. Epidemiologic studies have indicated that the most serious sequela occur in women are pelvic inflammatory disease (infection in the fallopian tubes) and ectopic

pregnancy (pregnancy in the tubes) (1). In addition, several reports have suggested that untreated maternal cervical chlamydial infection increases the risk of preterm delivery, premature rupture of membranes (PROM), and prenatal mortality or stillbirth (2). In newborns, infection can occur as a result of prenatal exposure; approximately 65% of babies born from infected mothers become infected during vaginal delivery. Infections caused by C. *trachomati*s are particularly difficult to confine as a high proportion of these infections are asymptomatic, thus making part of the population (those not tested) a reservoir for further transmission (3).

Mycoplasma genitalium (M. genitalium) which was discovered in the early 1980s has proved itself as a significant pathogen. It is similar to C. trachomatis in several respects such as preference for the genital tract, mode of transmission, making various adverse gynecologic and reproductive events and cervicitis (4). It has been implicated as an etiological agent of pelvic inflammatory disease (PID) independent of C. trachomatis and Neisseria gonorrhoeae (5). Also in one study, an association has been reported between M. genitalium and preterm delivery (6).

Screening of C. trachomatis with cell culture as a gold standard is very difficult and requires specimens from urogenital swabs, which are unacceptable to many people. Urine samples have cytotoxic effect and are not suitable for culture. Other routine tests for Chlamydia, such as enzyme immunoassays, have low sensitivity in asymptomatic individuals. Detection of M. genitalium by the culture method is also very time-consuming and requires up to 8 weeks for this microorganism whereas the nucleic acid amplification techniques can detect infectious agents in less than 8 hours (7).

Because of difficulties in cultivation of these fastidious organisms, some 15 years ago, a great deal of research has led to the development and evaluation of sensitive and specific diagnostic tests based on nucleic acid amplification tests. The important aspect of these assays was their capacity to be used on non-invasive specimens such as first void urine (FVU), which can be self-collected. Therefore it facilitates larger investigations on prevalence in many populations (8).

Nucleic acid amplification tests showed>95% sensitivity and specificity for detection of M. genitalium and C. trachomatis from urine samples (9). More recently, different PCR methods have been developed to detect STI-causing organisms. One of these methods is multiplex PCR (mPCR), which is a single step method that employs one tube containing PCR mixture and permits the simultaneous detection of more than one organism. This method has been used to detect multiple bacterial STIs in clinical specimens (10). This mPCR assay will be a more time-and cost-efficient diagnostic tool than the other currently employed techniques. Specificity and sensitivity of this method for detection of C. trachomatis and M. genitalium were estimated to be 100% and 98.9%, respectively (11). The aim of this study was to investigate the prevalence of C. trachomatis and M. genitalium, and related risk factors in pregnant women for the first time in Sabzevar city.

## MATERIALS AND METHODS

One hundred ninety-six urine samples of pregnant women (< 50 years old; in second or third trimesters of pregnancy) attending in the only maternity hospital (Mobini Martyr Hospital) of Sabzevar city, from September 2010 to February 2011 were screened for eligibility. Participants were ineligible if they had antibiotic use in the last 3 weeks, kidney disease, catheter usage, cognitive impairment, and illness severity that precluded study interview (12).

Two trained research assistants obtained the following information from each participant through a specific questionnaire and face-to-face individual interview: socio-demographic data (age, education, and residence), current history of clinical signs and urogenital symptoms (preterm labor, dysuria, urinary urgency, urinary frequency, vaginal discharge, vaginal itching) and sexual history (history of STIs, history of abortion, history of preterm delivery).

The research ethics board at Sabzevar University of Medical Sciences approved this research project and all participants signed a consent form.

10 ml of First Void Urine (FVU) were collected at least two hours after last urination in a sterile container. Samples were not frozen before testing but maintained at 4°C for one night in order to decrease inhibitors in urine (13). Finally, samples on ice were transported to the research laboratory. Immediately samples were centrifuged in 4°C (6000 g for 30min) and pellets were washed with PBS (Phosphate Buffer Saline) twice and prepared for DNA extraction by DNG-Plus kit (Cinnagen, Tehran, Iran) according to the manufacturer's protocol. Extracted DNAs were stored at −70°C (14).

**PCR Reaction**. Table 1 shows the sequences of primers used to amplify a specific 200 bp fragment of the Orf 8 gene of C. trachomatis and primers were used for a specific 346 bp fragment of the conserved part of MgPa adhesion gene (MG192) of M. genitalium (15).

**Table 1:** Primers used for duplex PCR.

Species	Sequences	
*C*. *trachomatis*	*Forward* 5′-CTAGGCGTTTGTACTCCGTCA -3′	200bp
	*Reverse* 5′-TCCTCAGGAGTTTATGCACT -3′	
*M*. *genitalium*	*Forward* 5′-AGTTGATGAAACCTTAACCCCTTG-3′	346 bp
	*Reverse* 5′-CATTACCAGTTAAACCAAAGCCT -3′	

Initially, to establish the conditions for Duplex PCR, we set up uniplex PCR using positive control DNA from C.trachomatis serovar L2 type strain 434/Bu (ATCC VR-902B) and M. genitalium type strain G37 (ATCC 35350). Amplification reactions were performed in a volume of 20 µl containing 1 µl of extracted DNA. The reaction involved one cycle at 94°C for 5 min, followed by 30 cycles of denaturation at 94°C for 30 sec, annealing at 54°C or 56°C for 35 sec, and extension at 72°C for 30 sec followed by one last cycle at 72°C for 8 min. The reactions were performed on a PCR system (Techne, UK).

Subsequently, duplex PCR conditions were established using a mixture of DNA from both control bacterial strains. The 40 µl Duplex PCR reaction mixture consisted of 1 µl from each extracted DNAs, 4 µl of the master mix solution, 0.5 µl of each specific oligonucleotide primer.

The reaction for the uniplex PCR was the same except that annealing temperature was 55°C. Once the Duplex PCR conditions had been optimized, the 196 FVU samples were subjected to Duplex PCR. In addition, negative controls lacking DNA and positive controls consisting 300 ng of genomic DNA from each bacterial strains were tested. The PCR products were analyzed by electrophoresis in the 0.5% TBE buffer through 1% agarose gel, stained with ethidium bromide, and DNA bands visualized using a UV transilluminator.

The collected data were entered into a computer using SPSS, version 15. The statistical relationship between the risk factors and infection were assessed using the chi-squared test.

## RESULTS

We recruited 196 pregnant women between September 2010 and February 2011 and 10 women (5% of total subjects) inevitably were excluded from the study. Subjects ranged in age from 17–45 years (mean 31.0 years); and a high proportion of women were below 30 years old which represented about 66.8% of all cases (Table 2). In this study, 74.4% of participants lived in the city and 65.8% of them did not have children. Also, 37.2% had at least one or more symptoms referable to the genito-urinary tract at time of interview. Vaginal discharge, urinary frequency and vaginal itching were common current clinical symptoms in all pregnant women.

**Table 2:** Statistical analysis of important variables in pregnant women.

Risk factors	Infected	%	Not-infected	%	Total	P-value
Age						NS
years old 30 >	22	11.2%	109	55.6%	131	
years old 30 ≤	9	4.5%	56	28.5%	65	
Education						NS
Illiterate/High school	24	12.2%	144	73.4%	168	
Diploma/university degree	7	3.5%	21	10.7%	28	
Symptoms						NS
Yes	11	5.6%	62	31.6%	73	
No	20	10.2%	103	52.5%	123	
Preterm Labor						P ≤ 0.05
Yes	4	2.0%	52	26.5%	56	
No	27	13.7%	113	57.6%	140	
History of STIs						NS
Yes	5	2.5%	19	9.6%	24	
No	26	13.2%	146	74.4%	172	
History of Abortion						P ≤ 0.1
Yes	3	1.5%	37	18.8%	40	
No	28	14.2%	128	65.3%	156	

NS: Not Significant

A statistical association was seen significantly between preterm labor and infection in this study (P-value ≤ 0.05). Also data analyses suggest a possible positive correlation of infection with history of abortion in pregnant women (P value ≤ 0.1). Of 196 women, 31 (15.81%) were positive in PCR test. C. trachomatis was detected in 27 samples (13.77%) and M. genitalium was positive in two samples (1.02%). Also co-infection was seen in two samples (1.02%).

Table 2 illustrates statistical analysis results of important variables in this study.

## DISCUSSION

There were several studies conducted in Iran that reported the prevalence of C. trachomatis to be from 2% to 11%. Only in one study 5.2% of pregnant women were reported to be infected with M. genitalium. It is worth noting that the diagnostic tests varied in the different studies and could contribute to the differences in the reported prevalence rates. Many studies have found that nucleic acid amplification tests are sufficiently sensitive to detect C. trachomatis and M. genitalium in first-void urine of women. Sensitivities have exceeded 95% in most studies when compared to detection with non-culture methods of endocervical specimens as a standard, while at the same time preserving high specificities (16).

The prevalence of C. trachomatis reported in this study (14.79%) is high in comparison with the moderate rates reported by other studies in Tehran, Iran (11.1% and 11.2% respectively) in similar study subjects and settings (17, 18). In two different studies conducted with Direct Immunoflourescent assay and ELISA consecutively in Bandarabbas and Ahvaz, south & south-west of Iran, the prevalence of C. trachomatis infection were reported to be 5.2% and 10% in pregnant women respectively (19, 20). In addition, two other studies from Tehran with similar methods (Direct Immunoflourescent and ELISA) report 2.7% and 2.9% Chlamydial prevalence in pregnant women (21, 22). C. trachomatis prevalence differs by treatment practices in different areas, but it seems that use of low sensitivity methods are the main reason for significant difference in prevalence rate reported from Tehran.

Depending on health status, the existence of symptoms and risky behavior of individuals, the prevalence of STIs in women differ from one country to another. Numerous surveys have been carried out to study the prevalence of C. trachomatis infection and risk factors among pregnant women or women attending antenatal clinics. C. trachomatis infection rate in our study is moderate in comparison to those found in several other countries: 10.1% in China, 10.5% in Saudi Arabia, 12.1% in Scotland, 35% in India (23–26).

In this study, M. genitalium was detected in 2.04% of participants, which illustrates a low percentage of occurrences in this region. In one study, performed by duplex PCR assay for simultaneously detection of M. genitalium and Ureaplasma Urealyticum in pregnant and non pregnant women of Tehran, the prevalence of M. genitalium was 5.2% (11/210) in total (27). The difference in the results of these two studies may be contributed to the type of subjects (pregnant vs. non-pregnant) and the sample type (urine vs. swabs). Also prevalence of M. genitalium in this study is similar to one study in Canada, with 3.6% infection in pregnant women (28).

Previous epidemiological investigations for C. trachomatis in pregnant women have revealed various associated risk factors for infection including age (in particular 18-27 years) and socio-economic status factors like urban residency or low-income (29). In this study, 75.8% of pregnant women with positive test results were less than 30 years old and 72.4% of infected women lived in the city, which confirmed results of previous research. Also in our study, a significantly higher detection rate of C. trachomatis infection was observed among primigravida (82.7%) compared to, multigravida (17.3%) and is similar to results of one study in Japan that demonstrated the prevalence of C. trachomatis was significantly higher among this group ( primigravida) of pregnant women (30).

Based on several studies, one of the leading causes of prenatal mortality is prematurity. Asymptomatic bacteriuria, gonococcal cervicitis and bacterial vaginosis are strongly associated with preterm delivery, but the role of C. trachomatis was less clear (31). Recently, new studies report that infections with C. trachomatis are associated with premature delivery in pregnancy and our findings confirmed this (32, 33).

In many studies, researchers have indicated C. trachomatis and infection caused by this microorganism as a risk factor in abortion, prenatal mortality and stillbirth (34). Although a weak association was seen in this study (Table 2), which confirmed this relationship, more studies with larger sample size in longer follow-ups are needed to investigate this factor during pregnancy and its outcome.

The rate of complications following a C. trachomatis infection is of crucial importance when evaluating cost-effectiveness in screening programs. Health care costs after a C. trachomatis infection in the United States have been estimated to exceed US $2 billion per year (35).

The Iranian health care system needs to revise the antenatal care offered and factors that encourage the utilization of antenatal services, such as offering the services free of charge, providing incentives, health promotion and adequate postnatal services.

## CONCLUSION

The prevalence obtained in this study is relatively high. In our region (east of Iran), there are no data about C. trachomatis and M. genitalium in pregnant women and this is the first study conducted in Sabzevar city. However, further studies with larger sample size and more focused on different groups at risk are needed for movement towards prevention and control of STIs.
